# When does perception facilitate or interfere with conceptual processing? The effect of attentional modulation

**DOI:** 10.3389/fpsyg.2012.00474

**Published:** 2012-11-02

**Authors:** Louise Connell, Dermot Lynott

**Affiliations:** ^1^Embodied Cognition Lab, School of Psychological Sciences, University of ManchesterManchester, UK; ^2^Embodied Cognition Lab, Decision and Cognitive Sciences Research Centre, Manchester Business School, University of ManchesterManchester, UK

Conceptual processing relies on the perceptual system, and, as such, perception affects conception. Many studies have demonstrated perceptual-conceptual interference, where perceptual stimulation in a particular modality leads to slower and/or less accurate conceptual processing of information from the same modality (e.g., Kaschak et al., 2005, 2006; Vermeulen et al., 2008). However, many other studies have demonstrated perceptual-conceptual facilitation, where perceptual stimulation leads to faster and/or more accurate conceptual processing in the same modality (Kaschak et al., 2006; van Dantzig et al., 2008; Connell et al., 2012; Connell and Lynott, in preparation).

At first glance, this apparent discrepancy seems like a serious problem for accounts of simulation-based concepts. Such theories hold that offline representations—that is, representations of objects and events that are not in the current environment (Wilson, 2002)—are functionally comprised of partial replays (i.e., simulations) of the neural activation captured during perceptual, motor, affective, and other experience (Barsalou, 1999, 2008; Glenberg and Gallese, 2012; Connell and Lynott, submitted). If conceptual representations therefore require modality-specific perceptual simulation, then why do they not consistently interact with perception? Why does perceptual stimulation sometimes impair and sometimes facilitate conceptual processing?

One account proposed that the difference lies in whether perceptual stimulation is concurrent with the conceptual task or precedes it, and whether or not the perceptual stimulus can be easily integrated into the simulation required by the conceptual task (Kaschak et al., 2005). According to this account, interference occurs when a concurrent perceptual stimulus cannot be integrated into the simulation required by the conceptual task. For example, Kaschak and colleagues argued that an upward-scrolling visual display could not be easily integrated with the sentence *The cat climbed the tree*, and hence interfered with its simulation. Facilitation occurs when a perceptual stimulus can be easily integrated into a simulation, regardless of whether the perceptual and conceptual components of the trial are presented concurrently or sequentially. For example, an image of a car would facilitate understanding a sentence like *The car approached you*.

However, this account cannot easily explain later findings. For example, concurrent tactile stimulation, in the form of vibrations to the palms and fingers, facilitates people's ability to judge the size of manipulable objects (Connell et al., 2012). Vibrotactile stimulation seems at least as distant from object representations of wallets and keys as upward-scrolling lines are from a cat climbing a tree. Yet, even though both perceptual stimuli appear “nonintegratible,” the former produced facilitation and the latter interference.

## Role of attention

We propose that these apparently discrepant effects can be resolved if one considers the attentional demands each task places on modality-specific processing. The perceptual and attentional systems are intertwined, and, since the conceptual and perceptual systems share modality-specific neural substrates, it should come as no surprise that they also share associated attentional mechanisms (e.g., Pecher et al., 2003; Connell and Lynott, 2010). Interference emerges when the perceptual stimulus occupies attention and leaves few resources free for simulation purposes. For example, a moving stimulus changes over time, and, as such, continuously captures attention in order to monitor its motion. Because a perceptual stimulus automatically directs exogenous attention toward that modality (e.g., Spence et al., 2001), processing a changing percept will wrest attention away from simulating in that modality and lead to interference effects. Conscious perceptual imagery, such as manipulation or memory rehearsal of perceptual information, will also occupy modality-specific attentional resources, and hence interfere with simulation in that modality.

In contrast, facilitation emerges when the perceptual stimulus directs attention toward a particular perceptual modality but leaves adequate resources free for simulation purposes. Selectively attending to a particular perceptual modality, even in the absence of a target, increases activation in the corresponding sensory cortex at the expense of other modalities (Foxe et al., 2005; Mozolic et al., 2008; Langner et al., 2011). That is, attention alone can pre-activate modality-specific perceptual systems so that subsequent target processing in that modality is facilitated. All else being equal, perceptual processing is hence faster in an attended than an unattended modality (Spence et al., 2000, 2001; Töllner et al., 2009).

In principle, both interference and facilitation can happen in concurrent and sequential presentation paradigms. For example, if a perceptual stimulus is presented concurrently with a conceptual task, it would interfere if it changes over time and continuously occupies attentional resources in that modality and would facilitate if it doesn't change and instead leaves that modality in an attentionally primed state. Similarly, if a perceptual stimulus has completed its presentation before a conceptual task, it would interfere if it is still occupying attentional resources and would facilitate if it no longer occupies attentional resources. Moreover, the sensory cortices are not homogenous, but rather contain some degree of feature specialization. In the visual modality, for instance, upward and downward motion are processed in different cell assemblies in the visual cortex (Mather et al., 1998), and attending to a particular direction of motion can increase activation in that direction-specific detector (Kamitami and Tong, 2006). As such, attentional effects can operate at either a whole-modality or a feature-specific level.

## Overview of effects

It is important, when disentangling facilitation and interference effects, to compare like with like. For that reason, we focus here on studies that (1) combine perceptual stimulation with a linguistic conceptual task and (2) measure responses to the linguistic conceptual task.

### Interference

A number of studies have shown interference effects because perceptual stimulation occupies attention in that modality, leaving insufficient resources for simulation.

In a concurrent paradigm, Kaschak et al. (2006: Experiments 1, 3) presented an auditory motion stimulus (i.e., an auditory illusion where the source of the sound appears to change location: upwards, downwards, towards or away) while participants read sentences onscreen that described auditory motion in a particular direction (e.g., *The jet pack roared into the sky*). People were slower to judge the sentences as sensible when they described the same direction of motion as the perceptual stimulus. Here, the motion in the perceptual stimulus meant that it continuously grabbed auditory attention as it changed over time. Auditory attention was therefore occupied in monitoring motion in a particular direction, and so there were insufficient attentional resources free when the sentence called for auditory simulation of motion in the same direction. Hence, the perceptual stimulus interfered with conceptual processing. The same account applies to Kaschak et al.'s (2005) studies of visual motion.

In a sequential presentation paradigm, Vermeulen et al. (2008) asked participants to first memorise auditory or visual stimuli (e.g., a series of visual shapes), then respond to a modality-specific property verification question (e.g., *lemon can be yellow*), and finally judge if another perceptual stimulus had been presented at the start of the trial. They found that property verification was slower when people held a perceptual memory load in the same modality. Here, although the perceptual and conceptual stimuli were presented in sequence, perceptual and conceptual processing effectively occurred concurrently because the memory load required imagistic rehearsal (i.e., conscious and effortful simulation) of the perceptual stimulus. In other words, the memory load task occupied modality-specific attentional resources, and so interfered with conceptual processing in that modality.

### Facilitation

Several other studies have shown facilitation effects because the perceptual stimulus directed attention to a particular perceptual modality without occupying resources.

In a concurrent paradigm, Kaschak et al. (2006: Experiments 2, 3) asked participants to listen to sentences over headphones that described auditory motion in a particular direction (e.g., *The jet pack roared into the sky*) while, in the background of the spoken sentence, an auditory motion stimulus was played. People were faster to judge that sentences were sensible when they described motion in the same direction as the auditory stimulus. Here, participants actually experienced two auditory stimuli: a perceptual stimulus of auditory motion and a speech stream delivering information for the linguistic conceptual task. Since the task goal of sensibility judgement required participants to listen closely to the sentence, their auditory attention was occupied by the speech stream and not by monitoring perceptual motion. As such, the perceptual stimulus directed attention toward motion in a particular direction, and hence facilitated simulation of auditory motion in that direction. These findings contrast with the interference effects found for auditory motion in the same paper when the sentences were presented in visual (text) form. When the perceptual motion stimulus is the only thing presented in that modality, attention will be occupied in monitoring its change over time, and simulation of same-direction motion in that modality will suffer from insufficient resources. But when the perceptual motion stimulus is presented in the same modality as a goal-relevant stimulus (i.e., something that requires a response, such as a sentence that must be judged as sensible or not), then the latter stimulus will have attentional priority. The perceptual motion will be perceived but not monitored—meaning it directs attention but does not continue to occupy it—and so simulation of same-direction motion in that modality will be easier (see also Zwaan and Taylor, 2006; Experiments 3, 5).

In a different concurrent paradigm, we stimulated people's hands or feet with tactile vibrations while asking them to compare the size of manipulable objects (e.g., *Which is bigger? wallet* or *key*: Connell et al., 2012). People were faster to name the relevant object when their hands were stimulated compared to their feet. Because the vibrotactile stimulation was constant and unchanging, it did not require monitoring and simply directed attention to the tactile modality in a somatotopic manner (i.e., the hand or foot area of the somatosensory cortex). Tactile stimulation to the hands therefore facilitated conceptual processing of objects whose simulations contained hand-related tactile information, while having no effect on objects whose representations did not include this information (e.g., *yacht*). The same effects emerged for proprioceptive stimulation. In other words, perceptual stimulation directed attention to modality-specific, body-specific systems and made simulation of such information easier.

Perceptual attention does not have to be directed by an exogenous stimulus but can also be endogenously directed as part of the implicit demands of a task. In recent work, we hypothesized that reading is, in effect, a concurrent paradigm of perceptual and conceptual processing. Both lexical decision and naming tasks involve recognition of visual word forms, and, as such, implicitly direct attention to the visual modality. Hence, we found that strongly visual words (i.e., referring to concepts with a strong visual component) have faster and more accurate lexical decision and naming times than weakly visual words, even when other variables such as length and frequency have been controlled (Connell and Lynott, in preparation). Furthermore, saying the same words aloud in a naming task, where the goal is correct pronunciation, also directs attention to the auditory modality. As a result, strongly auditory words are named more quickly and accurately than weakly auditory words. Indeed, such modality-specific attentional priming effects may be one of the main underlying reasons for concreteness effects in reading tasks (Connell and Lynott, 2012).

Finally, in a sequential paradigm, van Dantzig et al. (2008) asked participants to respond to a perceptual stimulus (visual light, auditory white noise, tactile vibration) and then to a property verification task (e.g., visual *broccoli is green*). People were faster to verify a property in the same modality as the preceding perceptual stimulus. Here, the perceptual stimulus directed attention toward its modality but did not require any further resources once the response was made, which meant that subsequent conceptual processing in that modality was facilitated (see also Vermeulen et al., 2009).

### What about action?

Similar combinations of facilitation and interference effects have been observed in studies of action and motor simulation, but we do not address them here because these studies tend to differ from those of perceptual simulation in one key respect. Perceptual simulation studies like those discussed above measure their dependent variable on a response act that is *unrelated* to the experimental manipulation (e.g., pushing a button, speaking aloud). In contrast, motor simulation studies typically involve measuring motor responses to action-related words and sentences (e.g., Glenberg and Kaschak, 2002; Boulenger et al., 2006; Zwaan and Taylor, 2006; Kaschak and Borreggine, 2008), and, as such, measure their dependent variable on a response act that is a *function of the experimental manipulation*. The net result of combining the manipulated and response modalities is to render it difficult to separate the effects of simulation on action from the effects of action on simulation, and to make the allocation of attentional resources susceptible to subtle differences in timing. By illustration, effects vary between interference and facilitation depending on the point in time that participants are made aware of the required action (Kaschak and Borreggine, 2008), the tense of verbs employed in the linguistic conceptual task (de Vega et al., 2004; Bergen and Wheeler, 2010), and the possibility of having to interrupt an action mid-execution (Boulenger et al., 2006). For these reasons, the picture of facilitation and interference effects in most motor simulation studies is more complex and variable than that in perceptual stimulation studies.
